# Proof-of-concept of harvest peak control using a strawberry cultivation emulator with artificial weather chambers

**DOI:** 10.1038/s41598-026-46422-z

**Published:** 2026-04-03

**Authors:** Hiroki Naito, Yasushi Kawasaki, Unseok Lee, Masaaki Takahashi, Fumiki Hosoi, Tomohiko Ota

**Affiliations:** 1 National Agricultural and Food Research Organization (NARO), Research Center for Agricultural Robotics, Tsukuba, Ibaraki 305–0856 Japan; 2 The University of Tokyo, Graduate School of Agricultural and Life Sciences, Tokyo, 113–8657 Japan

**Keywords:** Controlled climate system, Environmental control, Harvest date, Daily average temperature, Fruit ripening, Flowering date, Climate sciences, Ecology, Ecology, Plant sciences

## Abstract

Strawberry (*Fragaria × ananassa*) production requires a precise regulation of harvest-peak timing to meet market demand; however, fruit ripening remains sensitive to environmental fluctuations. We developed a peak-shift control system using a maturation simulator and conducted a proof-of-concept trial in climate chambers reproducing greenhouse conditions. Two control scenarios were designed: delayed flowering, wherein the predicted peaks lagged by a week and were corrected using heating offsets, and premature flowering, wherein the peaks advanced by a week and were adjusted using cooling offsets. Temperature offsets were explored within ± 5 °C, updated approximately twice weekly, to converge the predicted peaks with the target date on December 21, 2019. Results demonstrated successful alignment within ± 1 day in three of four treatments, surpassing previously reported prediction models. Cooling and heating treatments broadened and shortened harvest distributions by 2–3 and 2–4 days, respectively, suggesting the potential for balancing yield concentration. Post-harvest evaluation revealed no significant differences in morphology, grade distribution, or class proportion, although heating significantly reduced the soluble solid content, indicating a trade-off between accelerated ripening and sweetness. To our knowledge, this study provides the first experimental demonstration of simulation-in-the-loop, proactive harvest-peak control in strawberry, forming a basis for digital-twin-based cultivation control.

## Introduction

Strawberries (*Fragaria* × *ananassa*) are an important fruit crop worldwide. Advances in cultivation techniques, varieties, and management practices have enabled stable production systems that meet high global demand^1^. In Japan, strawberries are particularly popular during seasonal events such as the Christmas holidays^2^. Achieving early flowering and adjusting harvest timing to match peak demand are key strategies for maximizing market value^3,4^. However, because strawberry growth and ripening are highly sensitive to environmental factors, particularly temperature^5,6^, precise control of fruit maturation to align with target harvest dates remains difficult in open-field or semi-closed greenhouse conditions.

Traditionally, planting time and physical environmental control (heating, ventilation, shading) have been used to adjust harvest timing^7,8^. More recently, physiological knowledge of flowering and ripening has been incorporated into quantitative crop models^9–12^. For example, accumulated temperature (thermal time) models assume that fruit maturation occurs once a cumulative heat threshold (≈ 600 °C) is reached after flowering^11,12^. These models can predict maturation timing at the individual-fruit level but are limited in representing population-scale dynamics such as variation in flowering or ripening rates among fruits. Thus, despite progress in modeling crop physiology, quantitative and reproducible control of harvest peaks at the population level remains underdeveloped.

Harvest prediction research at the population level has advanced for several crops, including tomato, broccoli, apple, and persimmon^13–15^. A study targeted strawberry production in Florida and designed optimal yield distributions to enhance competitiveness with other regions^16^. A team at the University of Florida demonstrated the effectiveness of approximating early-season strawberry yield waves using a Gaussian distribution^17^.These studies mainly estimate yield distributions to optimize harvest timing or labor management, often using Gaussian or neural-network-based models. Similar approaches have been applied to strawberries, including modified versions of CROPGRO^18^ and photosynthesis-driven dry-matter production models^19^, which simulate cumulative yield with high accuracy. However, these models primarily serve predictive purposes validated at biweekly intervals and do not yet address dynamic control of harvest-peak timing.

In contrast, proactive harvest peak control studies remain scarce. For cucumbers, yield fluctuations have been leveled by staggering planting dates^7^, and in sweet pepper, dynamic yield “flushing” has been analyzed using crop models^20^. To our knowledge, no prior studies have quantitatively demonstrated active alignment of strawberry harvest peaks to specific target dates under controlled environmental conditions. Addressing this gap, the present study proposes a simulation-guided temperature control approach to achieve proactive harvest-peak regulation.

Recent technological advancements have led to the development of high-precision environmental control systems, such as artificial climate chambers, capable of accurately reproducing environmental factors, including temperature, humidity, light intensity, and CO_2_ concentration over time^21^. These systems enable high-fidelity emulation of hypothetical environments, making it possible to verify crop growth dynamics under tightly controlled conditions^22,23^. Although proof-of-concept studies on crop performance under reproducible environmental conditions are vital for the rapidly evolving field of digital-twin agriculture, such efforts in strawberries have so far focused mainly on robotics^24^, perception using synthetic datasets to train detectors^25^, and yield estimation^26^, rather than implementing closed-loop environmental control for harvest scheduling. This study extends digital-twin research toward proactive harvest scheduling through simulation-guided feedback control.

Therefore, in this study, we set out the following objectives:

- Assuming that the ripening speed of strawberries is primarily determined by temperature, we aimed to simulate the maturation process of fruits from multiple plants and develop a method for harvest peak control. To evaluate the practicality of the proposed algorithm, we used an artificial climate chamber capable of reproducing daily average temperatures with precision and examined the effects and limitations of peak-shift control.
- We also evaluated the effects of temperature-induced changes in maturation speed on fruit quality. Sudden temperature fluctuations or adjustments to ripening speed affect shipment suitability parameters, including sugar content, fruit shape, and external appearance^27^. Addressing the trade-off between control precision and quality preservation is a key factor in moving toward practical implementation.

Overall, this study demonstrates a simulation-guided, closed-loop approach for regulating harvest-peak timing under reproducible environmental conditions, providing one of the first empirical validations of proactive harvest-peak control in strawberry. Although prior research has been limited to individual fruit growth prediction models, few attempts have been made to scale these models to crop populations for actively controlling harvest peaks. We have addressed this limitation in our study. The present work extends this concept, linking a process-based maturation simulator with real-time temperature regulation to align population-level harvest peaks while assessing their quality impacts.

## Results

### Temperature reproduction and transition of offset values

**Fig. 1:**
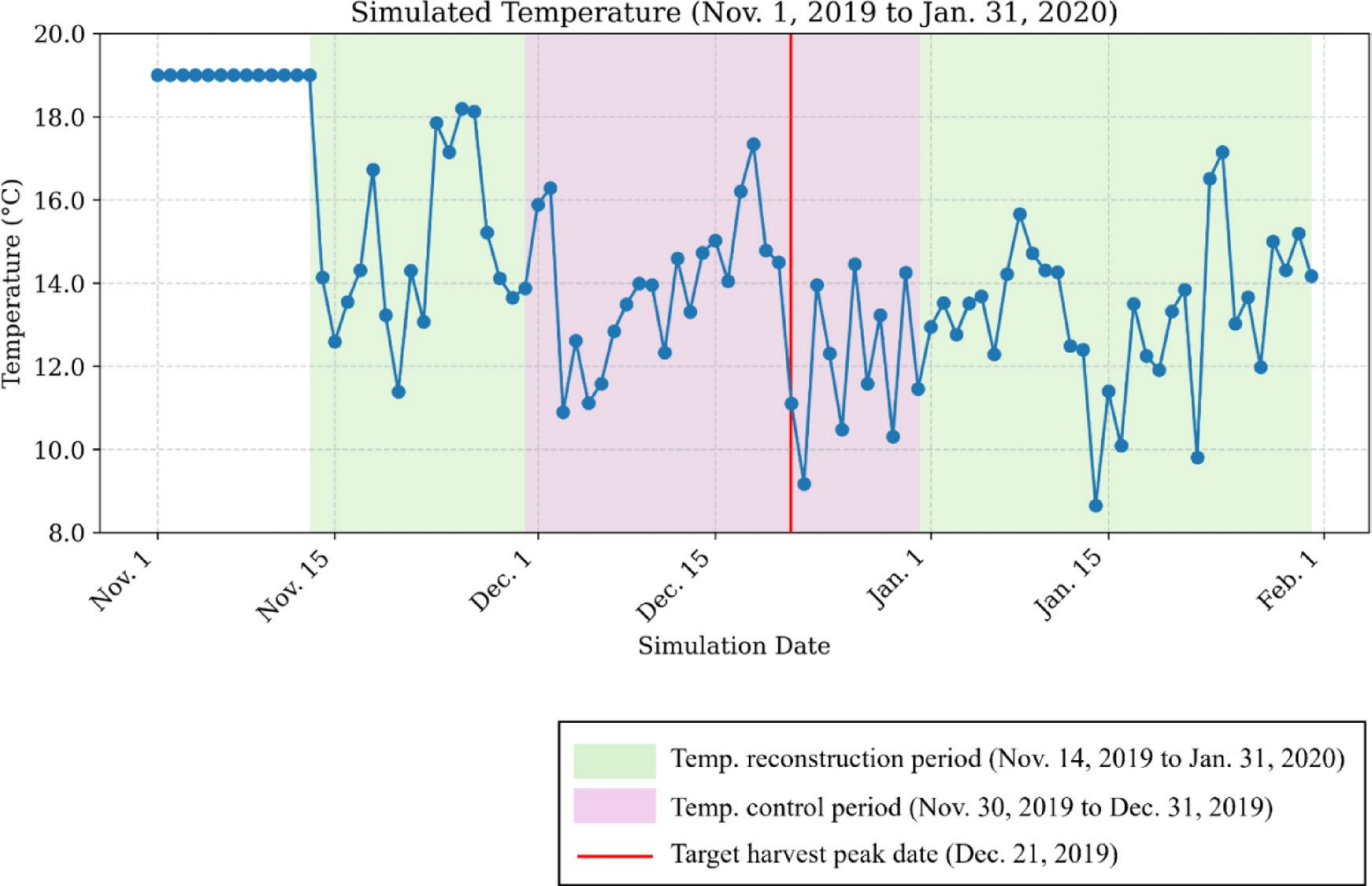
Simulated daily mean temperature profile in an artificial climate chamber capable of reproducing 2019 greenhouse conditions.

The daily average greenhouse temperature observed in 2019 in a high-eves greenhouse at the NARO Kyushu Okinawa Agricultural Research Center, Kurume Research Station, was reproduced using an artificial climate chamber (Fig. 1). From transplantation until November 14, the period during which flowering was promoted, the daily average temperature inside the chamber was fixed at 19 °C to induce the first flower bloom. From November 14 to January 31, the temperature in the chamber was reproduced based on the time series of observed values, ranging from 8.65 to 18.19 °C. During this period, the average temperature was 13.62 °C with a standard deviation of 1.99 °C, confirming consistency with the actual observed data.

Two experimental scenarios were designed to test the peak-shift control method:

1. *Premature-flowering (cooling, CL)*, wherein plants were assumed to flower approximately a week earlier than the reference schedule, and CL offsets were applied to delay fruit maturation; and.
2. *Delayed-flowering (heating, HT)*, wherein plants were assumed to flower approximately a week after the reference schedule, and HT offsets were applied to accelerate fruit maturation.

In both scenarios, the initial 1-week shift in the predicted harvest peaks was regulated such that maturation converged on the date of December 21. Each scenario was replicated twice (CL-1 and CL-2 for CL; HT-1 and HT-2 for HT). For each scenario, a corresponding control (CT) plot (CT-CL and CT-HT) was prepared without temperature offsets for direct comparison with the treated plots.

**Fig. 2:**
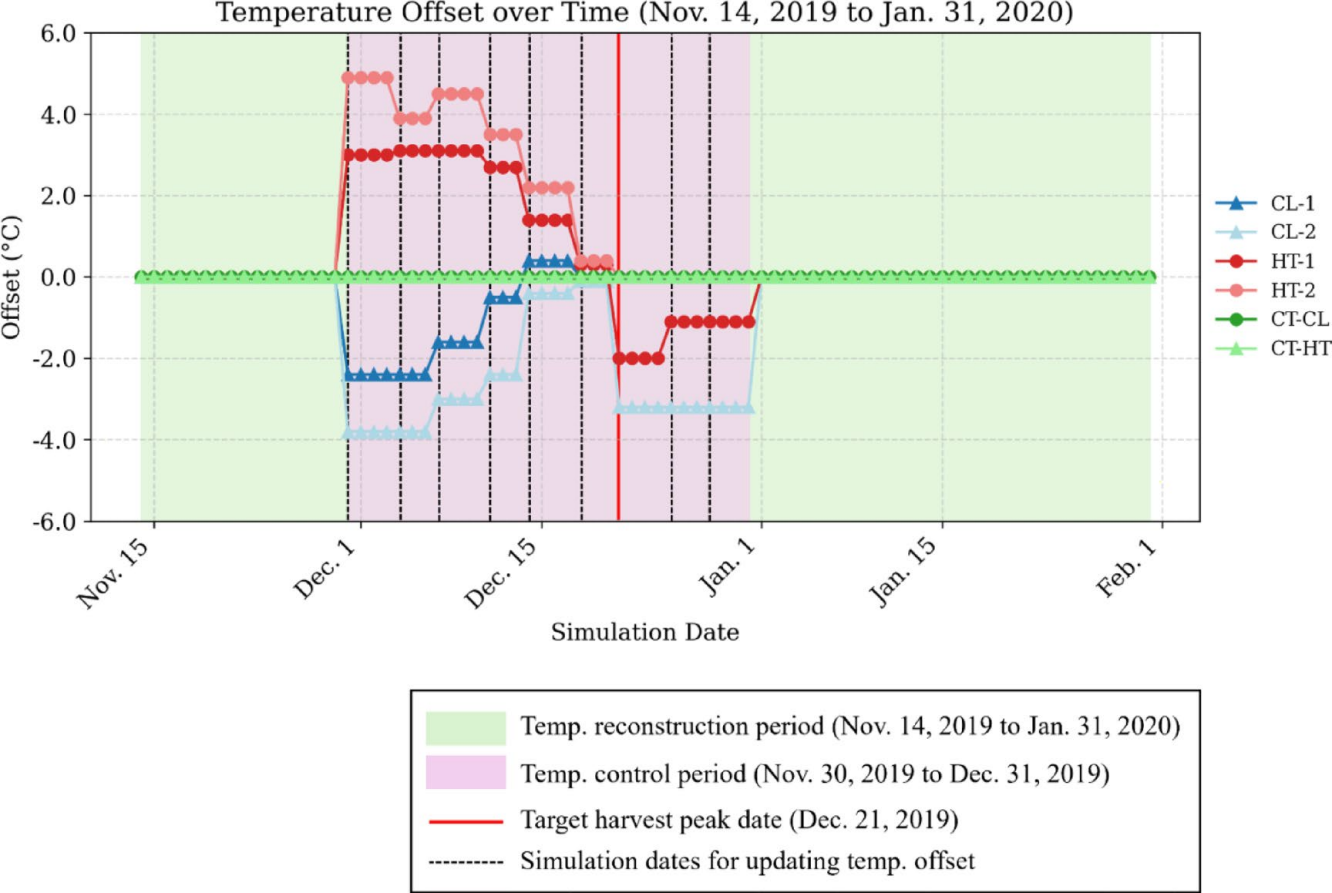
Time series of simulation-derived temperature offsets for the cooling scenario (CL, blue) and the heating scenario (HT, red), wherein harvest peaks were targeted a week earlier and later, respectively, relative to those obtained with the baseline reproduced temperature profile. Corresponding control (CT) plots (CT-CL and CT-HT, green) were cultivated without temperature offsets for comparisons with CL and HT, respectively.

Figure 2 shows the temporal transition of the temperature offset values calculated through simulation for each treatment. In the simulation, temperature offsets were derived to shift the predicted harvest peak date closer to the target of December 21. These offset values were added to the reproduced temperature data shown in Fig. 1 to regulate the peak shift. The blue line represents the CL group, the red line represents the HT group, and the green line represents the CT group in terms of daily average temperature. In the CT group, no offsets were applied, and the reproduced temperature was maintained; therefore, the offset remained constant at 0 °C.

However, temperature offsets were applied to the CL and HT groups starting from the onset of regulation on November 30. Although large corrections were required during the early phase of the regulation period, the offset magnitudes gradually narrowed. The offsets for all treatments converged to nearly 0 °C by December 18, ~ 3 weeks after the start of regulation. It was confirmed that in certain subplots, e.g., CL-2 and HT-1, relatively large offsets were reintroduced even after the target date. In the CL-2 plot, an offset as low as − 3.2 °C was applied, whereas in the HT-1 plot, the offset values of − 2.0 and − 1.1 °C were applied through the end of December.

## Transition of predicted harvest peak residuals and peak-shift effectiveness

**Fig. 3:**
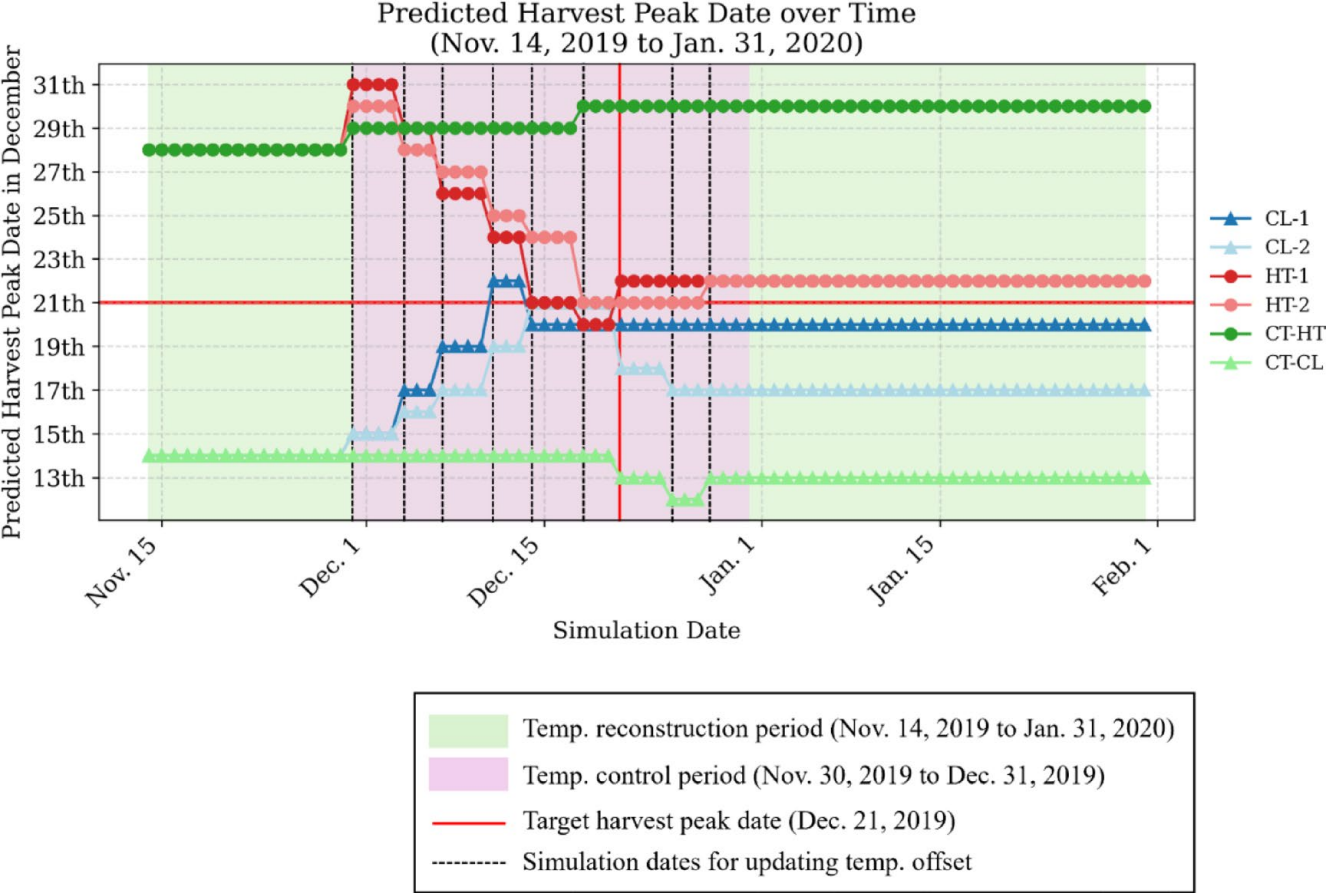
Time series of residuals between predicted and target harvest peak dates across simulation updates for the control, cooling, and heating treatments.

Harvest peak shifting was attempted in both experimental scenarios by applying temperature offsets. Figure 3 shows the temporal transition of residuals between the predicted harvest peak dates at each simulation time point and the target peak date (December 21). In the CT group (green), wherein no offset was applied, the predicted peak date remained relatively constant from November 14. In the CT-HT plot, the harvest peak was initially predicted to occur on December 28, and the actual observed peak occurred on December 30. Similarly, in the CT-CL plot, the peak was predicted to occur on December 14 and was observed on December 13, a day earlier. These results indicate that in the CT group—where no offset was applied—the approximately 1-week deviation from the target peak date was reproduced according to the initial scenario.

In the CL and HT groups, where offsets were applied starting on November 30, fruit maturation was appropriately regulated, and the predicted peaks (as indicated by markers in the figure) gradually converged toward the target date of December 21. As the residuals decreased, the absolute values of the temperature offsets also exhibited a decreasing trend (Figs. 2 and 3). In three treatment plots—CL-1, HT-1, and HT-2—the harvest peak successfully converged within ± 1 d of the target date. In the CL-2 plot, the residual improved to − 1 day (predicted peak on December 20) as of December 14, but simulations from December 21 onward showed a renewed advancement of the harvest peak. Consequently, the residual returned to − 4 days, with the peak occurring on December 17. Although attempts were made to suppress ripening through continued offset application after the target date in CL-2, the peak-shift regulation was not sufficiently effective, and the residual was not successfully corrected (Fig. 2).

**Fig. 4:**
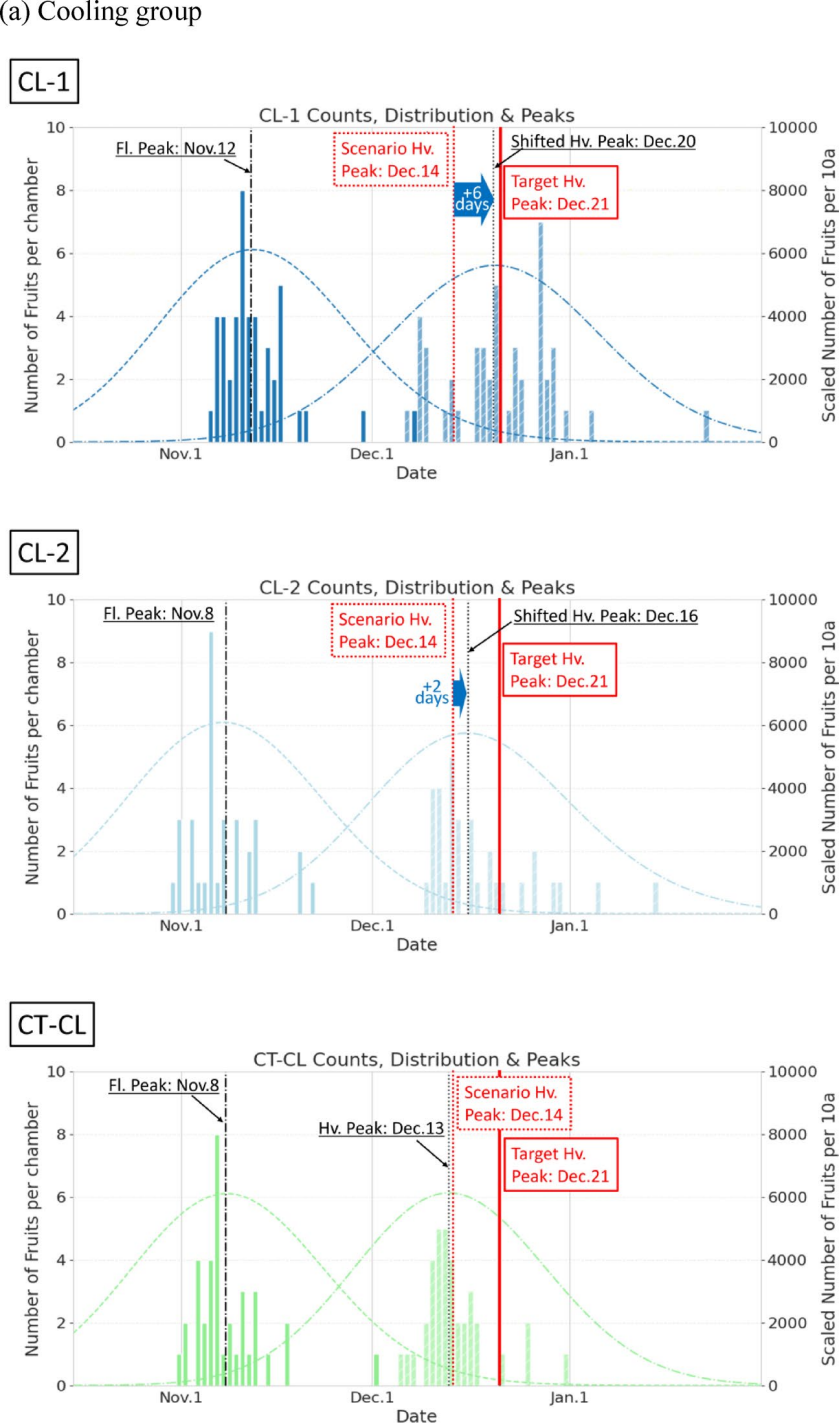

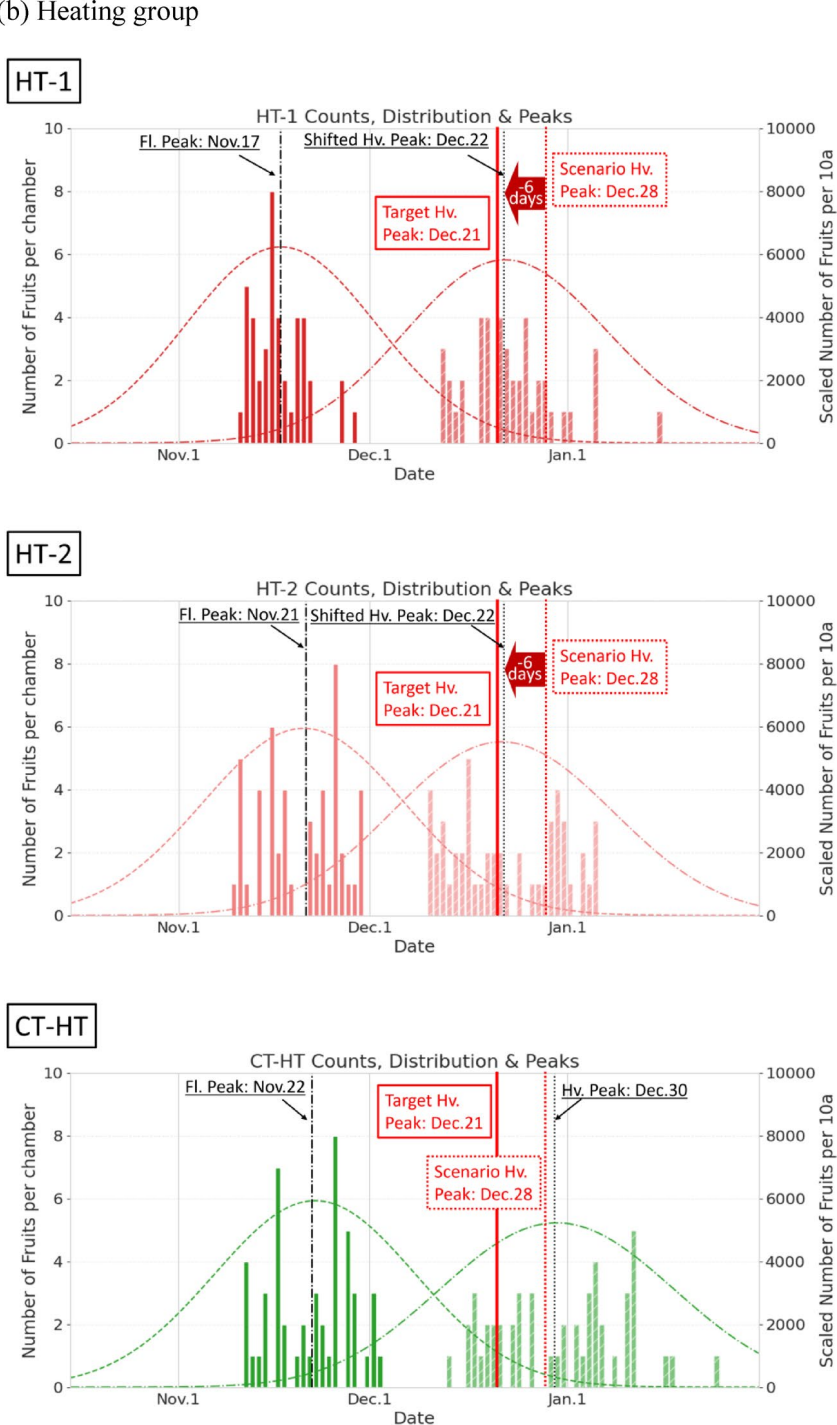
Comparison of flowering and harvest date distributions under the (**a**) cooling (CL) and (**b**) heating (HT) treatments versus their respective controls (CT-CL and CT-HT). The figure illustrates both peak shifts and changes in the shape of the harvest distributions. Vertical lines represent the different reference dates: Fl. Peak (flowering peak date), Scenario Hv. Peak (initially predicted harvest peak date under baseline reproduction without temperature offsets), Target Hv. Peak (target harvest peak date aimed for by applying temperature offsets), and Hv. Peak (finally observed harvest peak date).

Figure 4 presents the following comparative analysis of the effectiveness of harvest peak shifting in the CL and HT groups relative to their CT groups:

- *CL group*: In the CT-CL plot, early-flowering plants matured without delay, resulting in a harvest peak on December 13. However, the CL-1 plot experienced delayed maturation owing to the applied offsets, and the harvest peak shifted by + 6 d from the initial predicted date of December 14, reaching its peak on December 20. In the CL-2 plot, the absence of later-blooming flowers reduced fruit availability in the latter half of the harvest period, resulting in a minimal peak adjustment of approximately + 2 days. The treatment resulted in an earlier-than-expected peak on December 16, corresponding to a 5-day advancement relative to the target date.
- *HT group*: In the CT-HT plot, delayed maturation of late-flowering plants produced a harvest peak on December 30. In both the HT-1 and HT-2 plots, the application of HT offsets resulted in early maturation, with peak harvest on December 22. The residuals in these plots remained within + 1 day of the target.

In addition to the effects on harvest peak timing, changes in harvest duration were observed. The harvest distribution patterns revealed an extended harvest period with a broader distribution in the CL group owing to the suppressed ripening of later-developing fruits than in the CT (Fig. 4). In the HT group, the application of temperature offsets accelerated the harvest of later fruits, leading to a shortened harvest period and a slightly narrower distribution.

Table 1 summarizes the full-width at half maximum (FWHM) distributions of the flowering and harvested fruit counts. FWHM is an intuitive metric indicating the “spread” of a distribution, defined as the distance between two points on the *x*-axis where the curve reaches half of its maximum height. The FWHM values for the flowering period were almost identical between the CT and treatment groups, indicating similar flower count distributions (Table 1). During the harvest period, the CL group showed an FWHM extension of + 2 to 3 days relative to that in the CT, whereas the HT group exhibited a reduction in harvest duration of − 2 to − 4 days. These results demonstrated that temperature regulation enables harvest peak shifting while providing a means to manipulate the duration of the harvest period.

**Table 1:** Treatment-specific full width at half maximum (FWHM) for flowering and harvest dates.

Treatment	Flowering		Harvest	
	FWHM (days)	Difference of FWHM (days)	FWHM (days)	Difference of FWHM (days)
Premature flowering scenario (Cooling, CL)				
CL-1	35	0	38	+ 3
CL-2	35	0	37	+ 2
CT-CL	35		35	
Delayed flowering scenario (Heating, HT)				
HT-1	34	− 2	37	− 4
HT-2	36	0	39	− 2
CT-HT	36		41	

CT-CL and CT-HT: controls for CL and HT scenarios, respectively.

## Impact on fruit quality

Temperature-offset regulation during fruit ripening may impact quality. Post-harvest fruit quality was assessed using a standardized protocol, and comparisons were conducted between the CL and HT groups and their respective CT groups. The evaluation process was based on continuous indicators representing morphological and biochemical characteristics, as well as categorical indicators related to commercial grading standards.

## Effects of treatments on morphological and biochemical traits

Continuous quality indicators were classified as morphological (fresh fruit weight, diameter, and length) and biochemical traits (DM content and soluble solids content [SSC]). The normality of each variable was assessed using the Shapiro–Wilk test. For non-normally distributed data, the Mann–Whitney *U* test was used. Normally distributed data were analyzed using a one-way analysis of variance (ANOVA) and Tukey’s honest significant difference (HSD) test for multiple comparisons.

The results of the significance tests for the CL vs. CT and HT vs. CT groups are presented in Tables 2 and 3, respectively. No significant differences were observed in most traits. However, the HT group showed a statistically significant reduction in SSC compared with that in the CT group, which was the only trait that exhibited a statistically significant difference.

**Table 2:** Continuous quality metrics between cooling (CL) vs. control (CT) treatments.

Metric	CL (*n* = 76, mean ± SD)	CT (*n* = 80, mean ± SD)	Test	Statistic	*p*-value
Morphological traits					
Fresh weight (g)	18.54 ± 4.21	19.56 ± 6.56	Mann–Whitney *U*	*U* = 3,013.50	0.9266
Fruit diameter (max.) (mm)	35.45 ± 3.82	35.97 ± 4.31	Mann–Whitney *U*	*U* = 3,040.00	1.0000
Fruit diameter (min.) (mm)	33.30 ± 2.94	33.06 ± 3.30	ANOVA+Tukey’s HSD	*F* = 0.222	0.6378
Fruit length (mm)	34.81 ± 3.62	34.92 ± 4.80	Mann–Whitney *U*	*U* = 3,348.00	0.2755
Biochemical traits					
Dry matter ratio	0.1163 ± 0.0076	0.1172 ± 0.0118	Mann–Whitney *U*	*U* = 2,850.00	0.5017
SSC (Brix%)	10.53 ± 0.85	10.69 ± 1.29	ANOVA+Tukey’s HSD	*F* = 0.830	0.3638

ANOVA: analysis of variance; HSD: honest significant difference; and SSC: soluble solids content.

**Table 3:** Continuous quality metrics between heating (HT) vs. control (CT) treatments.

Metric	HT (*n* = 87, mean ± SD)	CT (*n* = 80, mean ± SD)	Test	Statistic	*p*-value
Morphological traits					
Fresh weight (g)	18.46 ± 6.22	19.56 ± 6.56	Mann–Whitney *U*	*U* = 3,095.00	0.2180
Fruit diameter (max.) (mm)	35.39 ± 4.28	35.97 ± 4.31	Mann–Whitney *U*	*U* = 3,251.00	0.4641
Fruit diameter (min.) (mm)	32.54 ± 3.60	33.06 ± 3.30	Mann–Whitney *U*	*U* = 3,125.50	0.2567
Fruit length (mm)	34.24 ± 4.70	34.92 ± 4.80	Mann–Whitney *U*	*U* = 3,219.00	0.4040
Biochemical traits					
Dry matter ratio	0.1178 ± 0.0133	0.1172 ± 0.0118	Mann–Whitney *U*	*U* = 3,602.50	0.6959
SSC (Brix%)	10.23 ± 1.25	10.69 ± 1.29	ANOVA+Tukey’s HSD	F = 5.307	< 0.05

SSC: soluble solids content; ANOVA: analysis of variance; and HSD: honest significant difference.

## Effects of treatments on grade distribution

The grade index reflects commercial shipping standards, with demand varying across classes (3 L, 2 L, L, M, and S) depending on timing and use. Tables 4 and 5 present the differences in grade distribution between the CL and CT and the HT and CT groups, respectively.

**Table 4:** Grade distribution and pairwise proportion *z* tests for cooling (CL) vs. control (CT) treatments.

Grade	CL (%)	CT (%)	z-score	p_raw	p_adj
3 L	10.0	21.7	–2.04	**< 0.05**	0.2082
2 L	45.0	28.9	2.13	**< 0.05**	0.1665
L	25.0	27.7	–0.39	0.6946	1.0000
M	17.5	19.3	–0.29	0.7698	1.0000
S	2.5	2.4	0.04	0.9703	1.0000

**Table 5:** Grade distribution and pairwise proportion *z* tests for heating (HT) vs. control (CT) treatments.

Grade	HT (%)	CT (%)	z-score	p_raw	p_adj
3 L	17.6	21.7	− 0.68	0.4953	1.0000
2 L	21.9	28.9	− 1.05	0.2930	1.0000
L	37.4	27.7	1.35	0.1754	0.8772
M	18.7	19.3	− 0.10	0.9202	1.0000
S	4.4	2.4	0.72	0.4733	1.0000

To assess distributional differences, *χ* tests were conducted to determine whether the overall grade distributions differed significantly. Subsequently, detailed pairwise comparisons between the grade categories were performed using two-proportion *z*-tests with Bonferroni cor^2^rection.

The results of the *χ*^2^ tests showed no significant differences in overall grade distribution between the CL-CT (*χ*^2^ = 6.54, *p* = 0.1625) and HT-CT (*χ*^2^ = 2.94, *p* = 0.5680) plots. No significant differences were observed in the individual grade categories after the Bonferroni correction. Although the uncorrected analysis results revealed significant differences (*p* < 0.05) in the proportions of the 3 L and 2 L grades between the CL and CT groups, these differences disappeared after Bonferroni correction. Thus, it can be concluded that, within the scope of this study, temperature offsets applied through the CL or HT treatments had no statistically significant impact on commercial-grade distribution ratios.

### Effects of treatments on class proportions

The class index was also evaluated. Class categorization was based on normal fruit enlargement, with fruits exhibiting sterility or deformities classified into relatively low ranks. Herein, the proportion of premium-grade fruits (designated KJ) among the total harvested fruits was defined as the “premium fruit ratio.” The sum of premium-grade (KJ) and standard-grade (B) fruit proportions was defined as the “marketable fruit ratio.” Differences between the groups were tested using two-proportion *z*-tests.

Tables 6 and 7 present the comparative results between the CL and CT groups and between the HT and CT groups, respectively. In both cases, there were no significant differences in premium fruit ratio or marketable fruit ratio. We concluded that, within the scope of this study, temperature offsets applied via the CL or HT treatments did not exert any significant impact on the class proportions critical for market distribution.

**Table 6:** Class proportions for cooling CL vs. control (CT) treatments.

Variable	CL (%)	CT (%)	z-score	*p*-value
Premium	88.75	85.54	0.61	0.5411
Marketable	95.00	96.39	–0.44	0.6627

**Table 7:** Class proportions for heating (HT) vs. control (CT) treatments.

Variable	HT (%)	CT (%)	z-score	*p*-value
Premium	83.52	85.54	–0.37	0.7124
Marketable	95.60	96.39	–0.26	0.7934

## Discussion

The peak-shift algorithm proposed in this study converged the harvest peak date to within ± 1 day of the target date (December 21) in three of four treatment plots (CL-1, HT-1, and HT-2). Notably, the reported ± 1 day accuracy is based on actual harvest counts, not solely on predictions derived from flowering records. In the simulation framework, the harvest dates were predicted based on the accumulated temperature and flowering date (Eq. 5); however, post-harvesting, the predicted dates were replaced with the observed harvest dates (Eq. 6). During the early regulation phase, peak prediction relied on flowering-derived forecasts, whereas in the later phase, the increasing proportion of fruits with observed harvest dates caused the prediction to converge toward the realized harvest distribution. This finding explains why the predicted harvest peak shifted close to the target date of December 21, with Fig. 4—showing the histogram of observed harvest dates—directly representing the realized peaks. The reported precision of ± 1 day precision reflects the regulation performance evaluated based on realized outcomes rather than purely forecasted peaks, demonstrating that a temperature offset search range of ± 5 °C, combined with twice-weekly update frequency, is sufficient to achieve effective harvest peak regulation. The resulting regulation accuracy of < 1 day surpasses that of previous prediction models, e.g., those predicted for persimmons with an error of ± 3 days^14^. Although differences in crop type, population scale, and experimental conditions complicate direct comparisons, the results highlight the potential of environmental regulation systems, e.g., artificial climate chambers, to modulate harvest timing. The CL-2 plot showed a deviation of − 4 days from the target date because of insufficient flowering in the latter half of the cultivation period, highlighting the fact that a sufficient and stable supply of floral buds is a prerequisite for effective peak-shift regulation. In cultivars or populations with inconsistent floral differentiation^28^, this regulation approach may not achieve the desired accuracy. To address this variability, dynamic and adaptive regulation is necessary to continuously monitor flowering trends and promptly update simulation scenarios on detecting anomalies.

A secondary effect of the temperature-offset treatment was observed on the harvest distribution shape (Table 1). Broader and narrower distributions were observed in the CL and HT groups, respectively. These outcomes likely stem from the greater impact of temperature adjustments on later-maturing fruits, which respond more strongly to environmental changes^6^. Such modulation of harvest spread may benefit strategic farm management, such as aligning yields with peak market demand or leveling labor requirements^7^. The quantitative contribution of ripening regulation to the changes in harvest distribution warrants further investigation to support its practical application.

Although the Python-based simulator could roughly estimate the population-level harvest peak based on limited sample plant data, several issues remain with the current regulation algorithms. During the early regulation phase, when large deviations from the target remained, the proportional feedback mechanism tended to produce relatively large temperature offsets. Such overcorrections—involving excessive heating or cooling—pose the risks of growth inhibition or substantial decline in fruit quality^29^. Increased energy consumption may result in economic losses^30^. Introducing advanced regulation strategies that optimize the temperature modulation across the entire regulation period is desirable to achieve convergence to the target peak date while minimizing the offset magnitude^31^. In the HT-1 and CL-2 plots, temperature offsets were applied even after the target date, as further adjustments to the timing of late-harvested fruits were attempted to shift the harvest peak. In the CL-2 plot, where the emergence of late flowers was delayed, the flowering prediction model may have failed to reproduce the actual phenological status, thereby reducing the effectiveness of the feedback-based correction. To address this limitation, the real-time monitoring of additional maturation indicators—e.g., fruit size or color metrics—can be incorporated into the feedback loop, enabling a more accurate and timely reflection of the ripening status in the simulation^32,33^. Applying temperature offsets beyond the target harvest date does not optimize yields for high-demand periods and may lead to unnecessary energy expenditure. Defining termination criteria and operational rules to optimize the regulation period is critical for improving future-generation regulation systems.

The observed reduction in SSC under heating is consistent with previous findings across open-field cultivation^34^, controlled-environment chambers^35^, and greenhouse systems^36^, all of which have reported significant decreases in strawberry sweetness in response to elevated temperatures. Among all quality indicators, only SSC exhibited a significant decrease in the HT group compared with that in the CT group. No significant differences were observed in other morphological, biochemical, or commercial grading metrics. The analysis supports that within the applied range of temperature offsets, the primary detectable effect was on sugar accumulation, whereas the impact on fruit size and visual quality remained limited. The significant reduction in SSC in the HT group reflected a trade-off between accelerated ripening caused by elevated temperatures and the maintenance of sugar content. This finding is consistent with prior reports that high temperatures reduce sugar accumulation in strawberries^29,37,38^. Soluble sugar content in strawberries is affected by day–night temperature differentials^36,39^. Enhancing the temporal resolution of temperature offsets—from daily to hourly—and lowering the nighttime temperatures to suppress the loss of respiratory carbohydrates can help mitigate this trade-off. Elucidating this trade-off is essential for developing operational strategies. For applications where sugar content is less critical, HT treatments may be a viable option, whereas for fresh-market strawberries, a cautious approach is required. No notable differences in quality indicators were observed between the CL and CT groups. A delay in harvest timing may pose the risk of reduced total yield. Prolonged fruit development periods—from flowering to harvest—because of lower temperatures, may reduce the harvest cycles within a season, leading to decreased yield per unit area and time^40^. When implementing low-temperature offset-based peak-shift regulation, the potential trade-offs between quality and total yield must be carefully considered.

In this study, we conducted a proof-of-concept using a small-scale artificial climate chamber, and a considerable gap remains before this approach can be scaled up for commercial operations.

1. Scalability and automation: To scale this approach to commercial greenhouses, key requirements include: (i) automated sensing of flowering and fruit status using camera-based detection of developmental stage, size, or color; (ii) closed-loop control linking offset calculations to climate computers for real-time implementation; and (iii) sampling strategies that extend from 7 to 14 plants to thousands, accounting for spatial variability. While temperature offsets were computed automatically, data collection and environmental adjustments were performed manually in this study. Integration of crop imaging, environmental sensors, and automated climate control systems into a unified pipeline would enable high-frequency, scalable regulation. To enable robust control at production scale, models must account for spatial heterogeneity in flowering^41^, supported by representative and distributed sampling strategies. The normal distribution–based models used in this study may be insufficient to capture such variability, underscoring the need for more flexible modeling approaches.
2. Robustness to flowering variation: The current modeling and control system assumes a certain degree of temporal predictability in flowering. However, real-world cultivation is subject to variability not only in the timing but also in the quantity of floral emergence, due to environmental and physiological factors. In our experiment, the CL-2 plot failed to produce sufficient late flowers, resulting in a breakdown of regulation. To address such risks, robustness can be enhanced by updating flowering curve estimates in real time, leveraging prior data from historical plots, and optionally incorporating sensing of fruit developmental stages to adjust predictions when flowering behavior deviates from expectations.
3. Controller design and termination rules: The current proportional feedback system occasionally reintroduced large offsets late in the cultivation period, leading to inefficient energy use with limited benefit. Introducing optimization-based or model-predictive control with cost terms for energy and fruit quality, along with explicit termination criteria—such as halting intervention after the target date unless benefits exceed a defined threshold—could improve both efficiency and stability.
4. Mitigating SSC penalties under high temperature: Since warmer ripening reduced SSC in this and previous studies^29,34–39^, peak-shift strategies must account for quality trade-offs. Time-of-day-aware control—such as applying positive offsets during the day while maintaining cooler night temperatures (~ 10–12 °C)—may help reduce respiratory sugar loss^36,39^. Implementing such set-point-aware regulation could mitigate SSC decline during heat-accelerated ripening.
5. Beyond Air Temperature Control: While temperature is a key driver of ripening, controlling air temperature alone may not fully optimize harvest timing and fruit quality. Multimodal environmental control—incorporating light (PAR), CO_2_, and humidity—offers a pathway to regulate both phenology and quality outcomes more precisely. For example, VPD-based humidification control (HT/D-VPDCS) has been shown to promote vegetative growth, accelerate first inflorescence harvest, and increase total yield in specific cultivars^42^. PAR and CO_2_ enrichment can accelerate sugar accumulation, promote fruit enlargement, and enhance overall quality, and their integration with thermal control may enable more balanced and efficient ripening. From a different perspective, previous research^43^ suggests that cumulative photosynthetic activity per leaf is a more accurate predictor of harvest timing than temperature alone, underscoring the need for alternative modeling approaches. In addition, approaches such as localized cooling^44^ or targeted ripening pauses^2^ remain promising options for fine-tuning peak control with minimal physiological stress. Beyond harvest timing, future models could incorporate predictions of post-harvest traits, e.g., fruit size and quality^45^. Expanding regulation beyond air temperature can enhance scheduling flexibility and stabilize fruit quality, but it also adds system complexity and interactions that can make control design more challenging.
6. Energy and economics: Practical deployment requires evaluating the cost-effectiveness of regulation strategies. A simplified energy–economic assessment showed that, during the high-demand period from the 1st week of December to the 2nd week of January, the cooling and heating treatments achieved 106.4% and 107.1% of baseline revenue, respectively. Daily revenue was estimated using price data from the Tokyo Metropolitan Central Wholesale Market together with standard strawberry production indicators published by a prefectural agricultural authority (6 t per 10 a, annual revenue of 5.4 million JPY). Additional energy costs were calculated using a fuel-consumption simulation model^46^ and an average A-heavy oil price of 107.4 JPY L^−1^. Even with these added energy costs, both treatments retained a positive margin relative to the baseline, suggesting potential economic feasibility when applied during premium demand periods. Incorporating such trade-offs into model predictive control frameworks that weigh market value against energy input^30^ would enhance both feasibility and profitability.
7. Generalizability: This study was conducted using only 7–14 plants of the June-bearing cultivar ‘Koiminori’ over a single season, limiting the statistical confidence and generalizability of the results. To contextualize the performance of the control algorithm itself, benchmarking peak-date errors against prediction-only baselines^17–19^ would further clarify the added value of feedback-based regulation beyond prediction accuracy alone. Cultivar-specific flowering responses are particularly relevant to peak-shift regulation. For example, readily inducible types such as ‘Kaorino’ and everbearing cultivars may respond differently to environmental conditions. Therefore, the effectiveness and flexibility of climate-based regulation may vary by genotype. To ensure robust and broadly applicable implementation, further validation across multiple cultivars and growing seasons is essential.

## Methods

### Cultivation conditions in artificial climate chambers

This study was conducted using the agro-environment (AE) emulator developed by the National Agriculture and Food Research Organization (NARO)^47^, which allows precise control of environmental factors such as temperature, humidity, CO_2_ concentration, and light. Two AE emulator-type artificial climate chambers (Emu-3HS; ESPEC MIC, Osaka, Japan) were used. Each chamber had internal dimensions of 1,720 mm (width) × 1,720 mm (depth) × 1,865 mm (height). The experiments were divided into three independent cultivation periods, defined from the transplantation date to the final fruit harvest date, as follows:

- Experiment 1: April 28, 2022 – July 19, 2022.
- Experiment 2: September 29, 2022 – January 24, 2023.
- Experiment 3: February 8, 2023 – May 22, 2023.

**Fig. 5:**
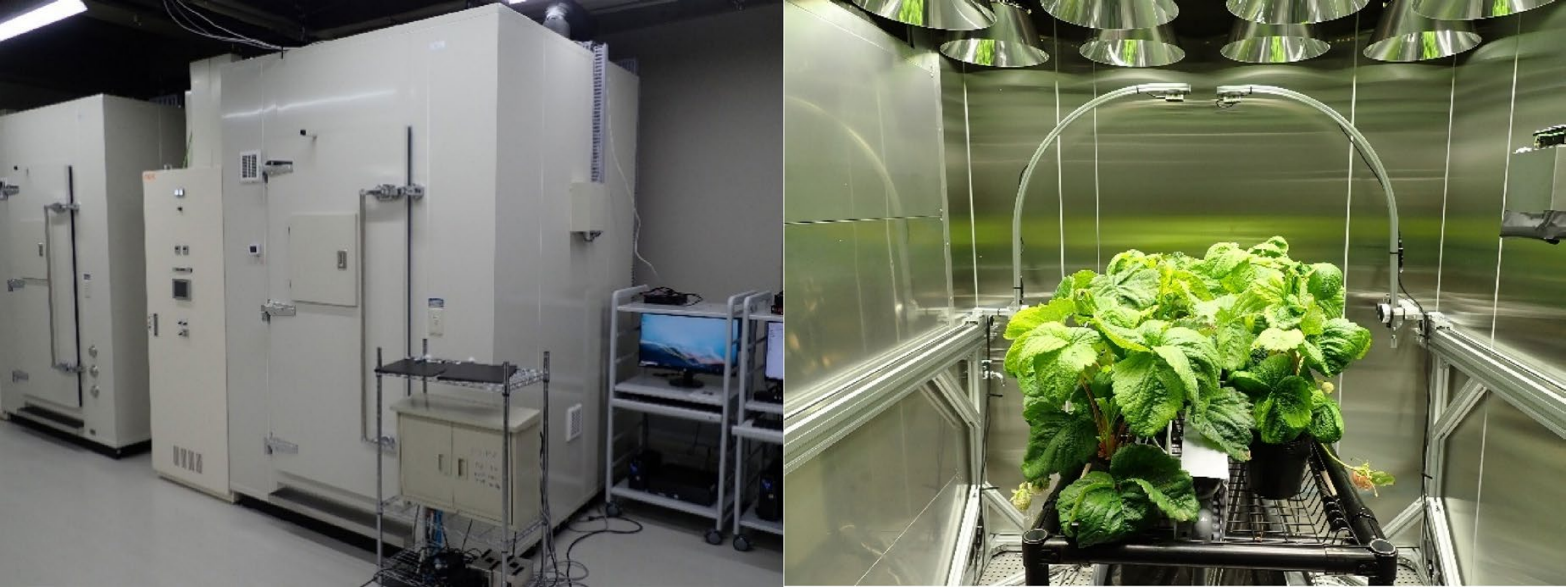
Photographs of the agro-environment (AE) emulator and strawberry cultivation setup (left: exterior view of the Emu-3HS chamber; right: interior view showing the ‘Koiminori’ strawberries planted in two rows on elevated benches).

Figure 5 shows the exterior of the climate chamber and the cultivation setup for the strawberry plants. Each chamber was equipped with a metal rack system that simulated an elevated hydroponic bench with an automated nutrient supply and drainage. The strawberry cultivar ‘Koiminori’ was planted in two rows of seven plants each (14 plants in total). The strawberry cultivar ‘Koiminori’ was bred by NARO and is registered as Ministry of Agriculture, Forestry and Fisheries Plant Variety Registration No. 28,183. Seedlings used in this study were propagated within NARO. In Experiment 1, ‘Koiminori’ was planted in both rows. In Experiments 2 and 3, only one side of each chamber (seven plants) was planted with ‘Koiminori’ and designated for the temperature regulation treatments.

Only the primary inflorescence (apical flower cluster) was harvested. A maximum of seven fruits were harvested per inflorescence, and subsequent flowers were manually removed. The number of leaves per plant was maintained at six throughout the experiment. Seedlings were pre-treated to induce floral differentiation, transplanted into 12-cm black pots, and spaced at 150 mm intervals. The growth medium consisted of a coconut coir substrate (Toyotane, BVB Coir Substrate for Strawberry, Aichi, Japan). Liquid fertilizer (OAT Agrio, Tokyo, Japan) was supplied at an EC of 0.8 dS m^−1^, with 300 mL applied per plant per day. Pollination was performed manually.

Environmental regulation was based on simulation outputs and was applied only to the air temperature. The other parameters were fixed at 70% relative humidity and 400 ppm. Illumination was performed using 16 LED plant-growth lights (HS180W/BGR001; ESPEC MIC) per chamber. This light emitted peak wavelengths at 399, 447, 575, 669, and 747 nm, with relative intensities of 33.7%, 100%, 67.6%, 73.5%, and 14.1%, respectively, simulating natural sunlight. The photoperiod was set at 12 h per day, with a light intensity of 500 µmol m^−2^ s^−1^. Air temperature, humidity, CO_2_ concentration, and light intensity were recorded at one-minute intervals using a dedicated regulation PC, allowing high-precision environmental replication.

### Acquisition of flowering and harvest date

Daily growth observations were conducted throughout the experiment to accurately record the flowering and harvest dates of the individual fruits. The criteria for defining each date are as follows:

- Flowering date: Defined as the first day on which the petals are fully open and before petal abscission begins.
- Harvest date: Defined as the day on which the entire fruit surface has achieved full coloration, indicating harvest maturity.

Upon confirming flowering, a tag was attached to the inflorescence. Each day, all fruits within the artificial climate chamber were visually inspected, and their developmental status was recorded. Harvesting was performed based on the degree of coloration, and the maturation period (in days) of each fruit was calculated from the recorded flowering and harvest dates indicated by the tag.

### Temperature reproduction and scenario design

The experiments were conducted over three independent periods (EXP1–EXP3) using two artificial climate chambers (Plant Emu-3 and Plant Emu-4) per period, resulting in six treatment groups. The treatment conditions applied to each group were as follows:

- HT group: Under a scenario in which the harvest peak was delayed by one week relative to the target date (December 21), a positive temperature offset was applied to accelerate ripening.
- CL group: In a scenario in which the harvest peak was advanced by one week, a negative temperature offset was applied to suppress ripening.
- CT group: This served as the baseline for the comparison between the HT and CL groups. No temperature corrections were applied, and the daily average greenhouse temperatures from October 2019 to January 2020 were accurately reproduced.

In the CT group, the daily average greenhouse temperatures observed at the Kurume Research Station of the NARO Kyushu Okinawa Agricultural Research Center were emulated with high fidelity. In the HT and CL groups, the reproduced temperature profiles were used as a base, and simulated temperature offsets were applied to align the predicted harvest peak with the target date.

Table 8 summarizes the conditions of the six experimental groups. In all cases, the start date for temperature replication was set as the virtual calendar date of November 14, 2019, and all subsequent regulation operations were managed based on this virtual calendar. The correspondence between the virtual and actual calendars was adjusted according to the flowering progression in each plot. Specifically, when more than half of the target plants reached the first flowering stage, a simulation was run, and the predicted harvest peak date was used to back-calculate the initial conditions of the pre-shift scenario. This method enabled the virtual alignment of “harvest peak attainment conditions” across different scenarios, allowing for comparative analysis of temperature regulation effects.

**Table 8:** Treatment conditions and real-to‐virtual calendar mapping for the six experimental plots.

Exp.	Exp. period (real calendar)	Chamber	Treatment	Treatment type	Predicted peak date before the treatment (virtual calendar)	Target peak date (virtual calendar)	No. of plants	No. of fruits
1st	Apr. 28, 2022 Jul. 19, 2022	Emu-3	CL-1	Cooling	Dec. 14, 2019	Dec. 21, 2019	14	46
		Emu-4	HT-1	Heating	Dec. 28, 2019	Dec. 21, 2019	14	43
2nd	Sep. 29, 2022 Jan. 24, 2023	Emu-3	HT-2	Heating	Dec. 28, 2019	Dec. 21, 2019	7	49
		Emu-4	CT-HT	Control (no heating)	Dec. 28, 2019	Dec. 28, 2019	7	49
3rd	Feb. 8, 2023 May. 22, 2023	Emu-3	CL-2	Cooling	Dec. 14, 2019	Dec. 21, 2019	7	33
		Emu-4	CT-CL	Control (no cooling)	Dec. 14, 2019	Dec. 14, 2019	7	36

### Simulation method for harvest peak prediction

Before implementing harvest peak regulation in the artificial climate chamber, predictions and analyses were conducted using a Python-based fruit maturation simulator. The simulation workflow was executed using the following steps:

### Preprocessing of environmental data

Time-series data of daily average temperatures observed at the plant factory of the Kurume Research Station, Kyushu Okinawa Agricultural Research Center, from October 1, 2019, to January 31, 2020, were imported from a CSV file. These data were linked to the date indices using the Pandas library in Python.

### Prediction and updating of flowering dates

The flowering dates for individual fruits were initially registered based on field observations, as described previously. For late-developing flowers that had not bloomed at the time of the simulation, the predicted flowering dates were estimated using a thermal accumulation model, with the first flowering date as the reference point. The estimation formula for unopened flowers is as follows:

The daily increment in bunch development $\:{d}_{\mathrm{b}}\left(t\right)$ was calculated as a function of the mean daily temperature $\:T\left(t\right)$, normalized by the cumulative temperature required for development from the first to the seventh flower $\:{CT}_{\mathrm{f}\mathrm{f}}$, and scaled to the maximum number of flowers per inflorescence ($\:{FLW}_{\mathrm{L}\mathrm{I}\mathrm{M}\mathrm{I}\mathrm{T}}=7$) using Eq. (1) as follows:

1 $$d_{b} \left( t \right) = \frac{{T\left( t \right)}}{{CT_{{ff}} }} \cdot (FLW_{{LIMIT}} - 1)$$

Here, $\:{CT}_{\mathrm{f}\mathrm{f}}$​ (252.1 ℃·day) represents the cumulative temperature required for the development from the first to the seventh flower. The cumulative development index was obtained using Eq. (2) as follows:

2 $$B\left( t \right) = \sum\limits_{{\tau = t_{0} }}^{t} {d_{b} \left( \tau \right)} ,$$

where $\:{t}_{0}$​ is the date of first flowering. The flowering date of the $\:j$-th flower ($\:j=2,\dots\:,7$) was defined as the first day when $\:B\left(t\right)$ reached or exceeded $\:j$ using Eq. (3) as follows:

3 $${\mathrm{Flowering}}\:{\mathrm{Date}}_{j} = \min \left\{ {t|B(t) \ge j - 1} \right\}$$

For fruits that had already flowered, the actual flowering date was calculated using Eq. (4). As previously noted, the number of fruits per inflorescence was limited to a maximum of seven, and flowering date predictions were restricted to the first seven fruits. Any additional flowers beyond the eighth were excluded from the simulation.

4 $${\mathrm{Flowering}}\:{\mathrm{Date}}_{j} = {\mathrm{Observed}}\:{\mathrm{Flowering}}\:{\mathrm{Date}}_{j}$$

### Prediction and updating of harvest dates

Similar to the flowering dates, harvest dates were either predicted or updated depending on the observation status of each fruit. For fruits that were not yet harvested, the harvest date was predicted using the cumulative daily mean temperature (CT) from the recorded or predicted flowering date. The number of days required from flowering to harvest was estimated using a stage-specific regression model, following the approach proposed in an earlier study^6^. In this model, the period from flowering to harvest was divided into four developmental stages (flowering, green, white, and turning), and the number of days required for each stage was predicted as a function of the average air temperature during that stage. The harvest date is obtained using Eq. (5) as follows:

5 $${\mathrm{Harvest}}\:{\mathrm{Date}}_{j} = {\mathrm{Flowering}}\:{\mathrm{Date}}_{j} + \sum\limits_{{i = 1}}^{4} {\widehat{{ND_{i} }}\left( {AT_{i} } \right)}$$

where the second term represents the predicted number of days from flowering to harvest. This duration is defined as the sum of stage-specific periods ($\:i$), each of which is calculated as a function of the average air temperature ($\:{AT}_{i}$) during the corresponding developmental stage^6^.

For fruit that had already been harvested, the predicted date was replaced with the actual observation date to improve the accuracy of the simulation using Eq. (6) as follows:

6 $${\mathrm{Harvest}}\:{\mathrm{Date}}_{j} = {\mathrm{Observed}}\:{\mathrm{Harvest}}\:{\mathrm{Date}}_{j}$$

### Estimation of harvest distribution and peak detection

Given the limited number of plants used in this proof-of-concept study (7–14 plants per chamber), the resulting harvest distributions were expected to be discrete and potentially ambiguous in terms of peak structures. To address this limitation, a Gaussian-based method was applied to estimate large-scale harvest distributions from the observed data. This approach was based on the assumption that the flowering and harvest dates of each fruit could be modeled as a normal distribution centered on the predicted harvest date. A similar method was reported in previous studies to approximate early-season harvest fluctuations using Gaussian distributions^17^.

Specifically, a sample normal distribution was generated for each fruit centered on its predicted harvest date. These distributions were then summed across all the fruits to produce a smooth, large-scale estimate of the total harvest distribution. The standard deviation of the Gaussian function (S.D. = 14.34 days) was derived from empirical measurements conducted in an experimental greenhouse. The cumulative result of this process produced a time series of normalized daily harvest volumes for all fruits.

The peak harvest date is defined as the date with the maximum estimated harvest distribution. The FWHM of the distribution was calculated to evaluate broadening or narrowing of the harvest period due to peak-shift interventions. The FWHM was defined as the number of days between the earliest and latest dates at which the distribution remained above half of its maximum value, applied to both flowering and harvest count distributions.

### Peak-shift algorithm via temperature offset

**Fig. 6:**
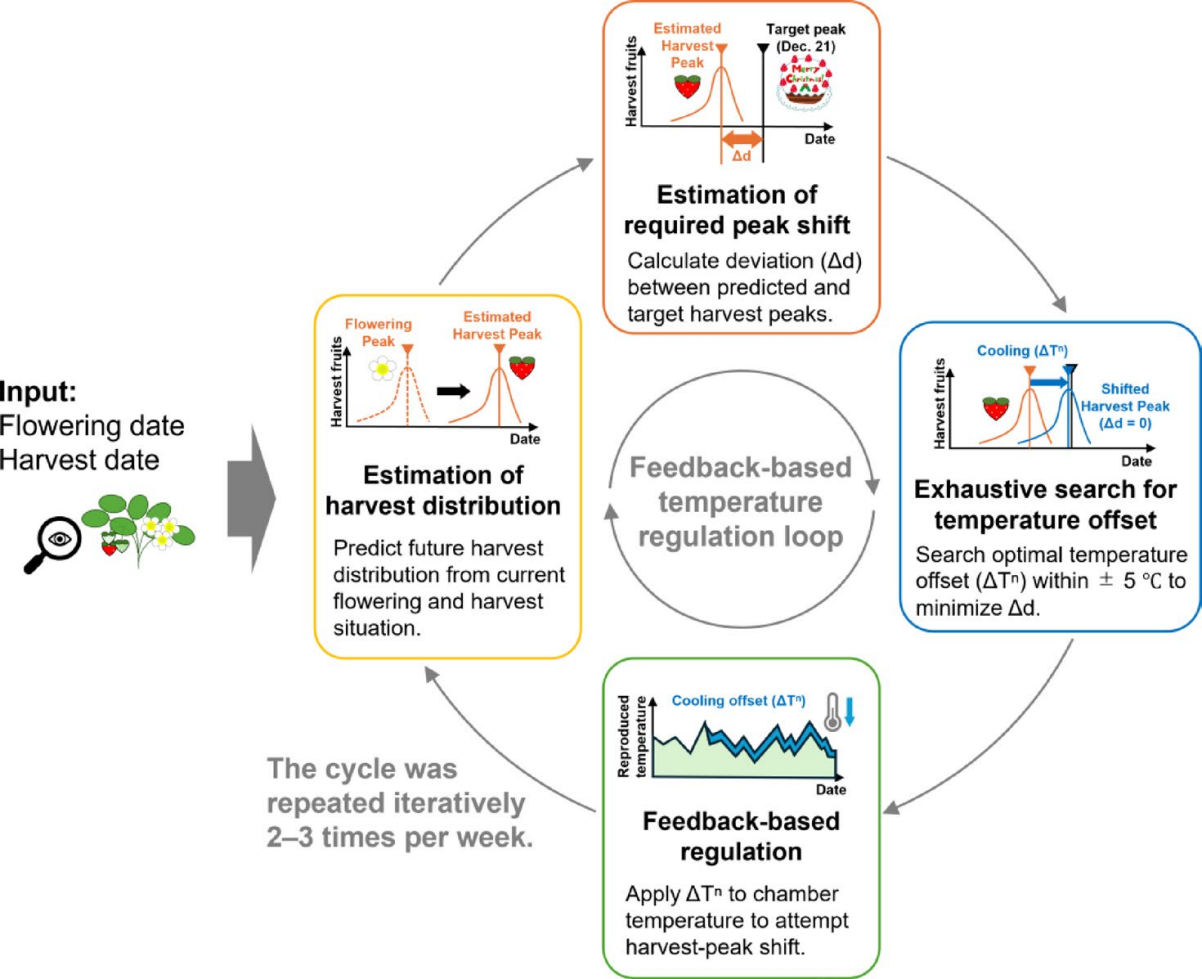
Feedback-based temperature regulation loop for peak-shift control. Schematic overview of the feedback framework aligning the harvest peak with the target date (December 21, virtual calendar). At each update, the model predicts the harvest peak, calculates the deviation (Δd), and searches for the optimal temperature offset (ΔT^n^ ± 5 °C) to minimize it. The selected offset is applied to the chamber, and the loop is iteratively updated two to three times per week.

In both CL and HT groups, feedback regulation linked to the simulation outputs was implemented to align the harvest peak with the target date of December 21 (on a virtual calendar). The regulation algorithm consists of the following steps (Fig. 6):

### Estimation of required peak shift

The difference Δd (in days) between the predicted harvest peak and the target date was calculated at each simulation time point.

### Exhaustive search for temperature offset

A range of candidate temperature offsets ΔT^n^ from − 5 to + 5 °C (in 0.1 °C increments) was exhaustively tested to identify the regulation temperature that would achieve Δd = 0. The search range was determined based on the optimal temperature range for strawberry growth. When multiple candidate offsets satisfied the condition, the value with the smallest absolute offset (|ΔT|) was selected.

### Feedback-based regulation

The calculated offset ΔT^n^ was added to the baseline reproduced temperature, and the resulting temperature was applied to shift fruit maturation either forward or backward, thereby attempting to realign the overall harvest peak.

Simulations for peak prediction began a month before the target date of November 21 (virtual calendar). The initial temperature regulation intervention was initiated on November 30, three weeks before the target. Simulations to update the offset temperature were conducted at approximately twice-weekly intervals (2–3 times per week), specifically on 4, 7, 11, 14, 18, 21, 25, and December 28 (all dates according to the virtual calendar).

#### Evaluation of the impact on fruit quality

To comprehensively assess the effects of temperature offset–based harvest peak shifting on both external and internal fruit quality, postharvest quality evaluation was conducted on all harvested fruits. The assessment included both continuous indicators—representing morphological and biochemical traits—and categorical indicators relevant to market shipment standards.

### Morphological continuous indicators

- Fresh weight (g): The mass of each fruit was measured immediately after harvest using an electronic balance (accuracy, 0.01 g).
- Maximum diameter (mm): The largest transverse diameter of the fruit was measured using a digital caliper (accuracy 0.1 mm).
- Minimum diameter (mm): The smallest transverse diameter of each fruit was measured in the same manner.
- Fruit length (mm): The length from the base to the tip of the fruit was measured using a digital caliper (accuracy 0.1 mm).

#### Biochemical continuous indicators

- DM ratio (%): Fruits were dried in a constant-temperature oven at 80 °C for three days, and dry weight was measured. The DM ratio was calculated as follows:

7 $$DM\:{\mathrm{Ratio}}\left( \% \right) = ({\mathrm{Dry}}\:{\mathrm{Weight}}/{\mathrm{Fresh}}\:{\mathrm{Weight}}) \times 100$$

- SSC (Brix%): SSC was measured using a digital refractometer (PAL-BX|ACID4; Atago Co., Ltd.).

#### Categorical indicators related to shipment standards

- Grade: Fruit grade was determined based on fresh weight in accordance with thresholds commonly used in major production regions for the cultivar ‘Koiminori. ’ Specifically, fruits were classified as follows: 3 L (> 23 g), 2 L (> 18 g), L (> 14 g), M (> 10 g), S (> 6 g), and nonstandard (≤ 6 g).
- Class: This indicator reflects the visual quality of fruit appearance. Proper fruit enlargement is a key factor in classification, whereas sterile or malformed fruits are ranked lower. The class was categorized into KJ (premium), B (standard), and N (non-standard). The following two indicators were calculated based on these categories:

8 $${\mathrm{Premium}}\:{\mathrm{Fruit}}\:{\mathrm{Ratio}}\left( \% \right) = \frac{{{\mathrm{Number}}\:{\mathrm{of}}\:KJ\:{\mathrm{Fruits}}}}{{{\mathrm{Number}}\:{\mathrm{of}}\:{\mathrm{Total}}\:{\mathrm{Fruits}}}} \times 100$$

9 $${\mathrm{Marketable}}\:{\mathrm{Fruit}}\:{\mathrm{Ratio}}\left( \% \right) = \frac{{{\mathrm{Number}}\:{\mathrm{of}}\:KJ\:{\mathrm{Fruits}} + B\:{\mathrm{Fruits}}}}{{{\mathrm{Number}}\:{\mathrm{of}}\:{\mathrm{Total}}\:{\mathrm{Fruits}}}} \times 100$$

Statistical comparisons were conducted between the CT and CT groups, and between the HT and CT groups for all indicators. For continuous indicators, the normality of each variable was evaluated using the Shapiro–Wilk test. If normality was confirmed, one-way ANOVA and Tukey’s HSD tests were performed. For non-normally distributed data, the Mann–Whitney *U* test was used.

Regarding the grade (size category), differences in overall distribution were assessed using the *χ*^2^ test. Pairwise comparisons between grade categories were conducted using two-proportion *z*-tests with the Bonferroni correction.

For class-related indicators—premium fruit ratio and marketable fruit ratio—two-proportion *z*-tests were used to compare the ratios across treatment groups. All statistical analyses were performed using the statsmodels library in Python 3.9.

## Data Availability

The dataset and code presented in this study are available on request from the corresponding author.
